# The potential benefit of artificial intelligence regarding clinical decision-making in the treatment of wrist trauma patients

**DOI:** 10.1186/s13018-024-05063-6

**Published:** 2024-09-19

**Authors:** Marco Keller, Meret Rohner, Philipp Honigmann

**Affiliations:** 1 Hand and Peripheral Nerve Surgery, Department of Orthopaedic Surgery and Traumatology, Kantonsspital Baselland (Bruderholz, Liestal, Laufen), Bruderholz, Switzerland; 2 Medical Additive Manufacturing Research Group (MAM), Department of Biomedical Engineering, University of Basel, Allschwil, Switzerland; 3 Department of Biomedical Engineering and Physics, Amsterdam UMC, University of Amsterdam, Meibergdreef 9, Amsterdam, The Netherlands; 4 Medical Faculty, University of Basel, Basel, Switzerland; 5 Hand and Peripheral Nerve Surgery, Department of Orthopaedic Surgery, Traumatology and Hand Surgery, Spital Limmattal, Schlieren, Switzerland

## Abstract

**Purpose:**

The implementation of artificial intelligence (AI) in health care is gaining popularity. Many publications describe powerful AI-enabled algorithms. Yet there’s only scarce evidence for measurable value in terms of patient outcomes, clinical decision-making or socio-economic impact. Our aim was to investigate the significance of AI in the emergency treatment of wrist trauma patients.

**Method:**

Two groups of physicians were confronted with twenty realistic cases of wrist trauma patients and had to find the correct diagnosis and provide a treatment recommendation. One group was assisted by an AI-enabled application which detects and localizes distal radius fractures (DRF) with near-to-perfect precision while the other group had no help. Primary outcome measurement was diagnostic accuracy. Secondary outcome measurements were required time, number of added CT scans and senior consultations, correctness of the treatment, subjective and objective stress levels.

**Results:**

The AI-supported group was able to make a diagnosis without support (no additional CT, no senior consultation) in significantly more of the cases than the control group (75% vs. 52%, *p* = 0.003). The AI-enhanced group detected DRF with superior sensitivity (1.00 vs. 0.96, *p* = 0.06) and specificity (0.99 vs. 0.93, *p* = 0.17), used significantly less additional CT scans to reach the correct diagnosis (14% vs. 28%, *p* = 0.02) and was subjectively significantly less stressed (*p* = 0.05).

**Conclusion:**

The results indicate that physicians can diagnose wrist trauma more accurately and faster when aided by an AI-tool that lessens the need for extra diagnostic procedures. The AI-tool also seems to lower physicians' stress levels while examining cases. We anticipate that these benefits will be amplified in larger studies as skepticism towards the new technology diminishes.

## Introduction

Conventional x-ray imaging is the cornerstone of fracture detection in emergency care units.

Decisions regarding further management rely on details revealed in the X-ray, including the presence of a fracture, any displacement, or involvement of the joint surface. Up to now, radiographs are usually interpreted by humans, mostly radiologists. However, several studies indicate a high number of diagnostic errors in radiograph interpretation with real-time error-rates of up to 3–5% in daily practice. [1–4]

Artificial intelligence (AI) is spreading in the medical field. Deep learning (DL) models are successfully used to detect skin cancer from photographs, breast cancer from mammography images, lung cancer from computed tomography (CT) scans or to identify diabetic retinopathy from eye images. [5–8] In many of the mentioned fields, DL models achieve the accuracy of an expert.

The use of DL models for automated fracture detection in conventional radiography has been explored by several studies. The literature shows that DL models can reliably detect fractures in the upper limb, the lower limb, and the spine. According to some authors a paradigm shift in fracture detection can be observed currently. [9]

Even though many studies highlight the superiority of AI-enabled fracture detection models, the integration into clinical routine remains scarce. A potential barrier to embracing these technologies could be the absence of evidence in existing research connecting the use of AI tools to its benefits.

The aim of this study was to investigate how a reliable AI-enabled distal radius fracture detection model substantially influenced the treatment of wrist trauma patients in an emergency care unit. We intended to not only uncover potential effects on doctors’ decision-making process and patient’s outcomes, but also to quantify these effects.

## Material and methods

To investigate the potential impact of an AI-based fracture detection model we designed an experiment with a special focus on recreating a close-to-reality scenario in an emergency care unit. Two groups of physicians were confronted with 20 virtual, but realistic cases of wrist trauma patients. The task of the participating doctors was to assess clinical information and radiographs, find the correct diagnosis and possibly provide a treatment recommendation according to the usual workflow in the hospital’s emergency care unit.

Throughout the experiment, one group of physicians was supported by an AI-enabled fracture detection model while the other group was not. The participating physicians were volunteering residents recruited at one regional hospital site. All participants were either residents at the emergency care unit or at the orthopaedic department and gave written informed consent to participate in the study. A description of the participants’ characteristics is available in Table 1. Participants in Group A were slightly older and had more clinical experience.

**Table 1:** Participants’ characteristics

Characteristic	Group A (AI support)	Group B (no AI support)	*p* value
Physicians in total	12	10	
Orthopedic physicians	6	5	
Emergency physicians	6	5	
Female/male	5/7	4/6	
Average age in years	31.3 ± 2.5	28.3 ± 1.3	0.002
Previous training in months			
Orthopedic surgery	18.5 ± 21.8	15.0 ± 23.2	0.72
General surgery	7.8 ± 12.6	2.9 ± 7.6	0.30
Internal medicine/geriatrics	11.3 ± 13.0	4.3 ± 5.1	0.13
Emergency medicine	8.0 ± 9.3	2.7 ± 3.6	0.10
Radiology	0 ± 0	0 ± 0	–
Clinical medicine	45.5 ± 21.0	24.9 ± 19.4	0.03

Numbers are means; ± standard deviations

This study was approved by the ethics committee of Northwestern and Central Switzerland (EKNZ) (BASEC-Nr. 2023-01293). Our report follows the guidelines of the “CONSORT-AI extension” of the CONSORT 2010 statement, which is a reporting guideline for clinical trials evaluating interventions with an AI component. [10]

### Fracture detection model

The DL model used in the present experiment was developed by our study group within another research project. [11] It is based on state-of-the-art machine learning methods used for object detection and was trained on our own dataset with the objective to detect and localize distal radius fractures in the dorso-palmar projection of conventional wrist radiographs. In an experimental environment our best model achieved a diagnostic accuracy of 98.5% and an AUC of 0.995 (corresponding to a sensitivity of 0.987 and a specificity of 0.987) regarding the classification task (“fracture present or not”).

**Fig. 1:**
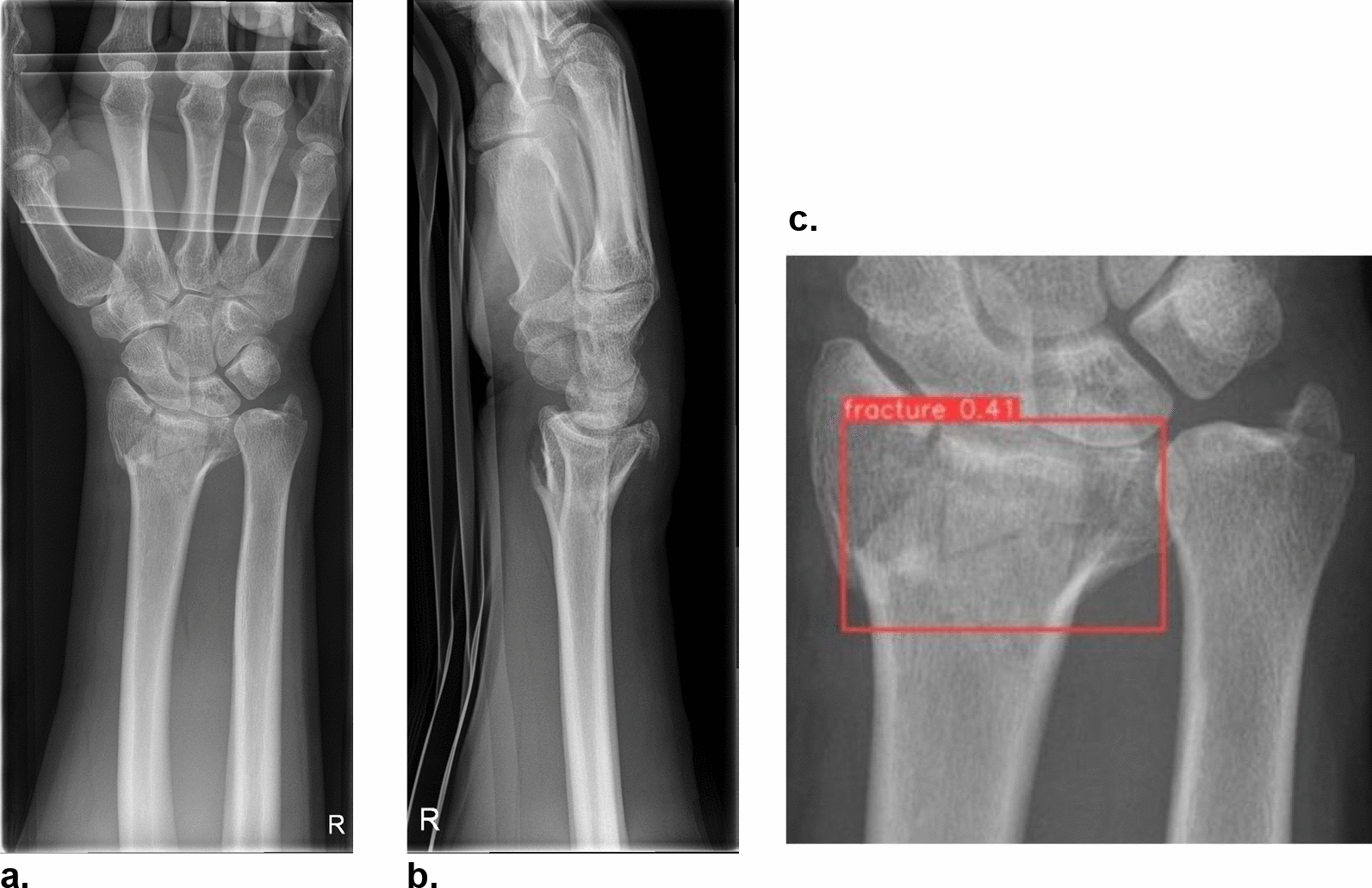
Radiographs showing one example of a distal radius fracture used for a virtual patient case. **a** Dorsopalmar wrist radiograph. **b** Corresponding lateral projection. **c** Dorsopalmar wrist radiograph with a bounding box added by the AI-enabled fracture model marking the presence and location of a distal radius fracture. Only physicians from group A were provided with this image.

The participants of our study who were allocated to group A were given the information that they are supported by “an artificial intelligence tool which detects distal radius fractures with a diagnostic accuracy of 98.5%”. This information was based on the performance metrics of our own distal radius fracture detection model and corresponds with similar models found in the literature. [12–17] The wrist radiographs from the patient cases, in which a fracture was present, were therefore augmented with a bounding box, clearly marking the presence and location of the distal radius fracture. (Fig. 1).

### Experiment setup

The experiment took place in a Level II trauma center in a regional hospital in the northwestern part of Switzerland with a high number of trauma patients presenting to the emergency department.

All participants were initially randomly allocated to group A (AI-supported) or group B (no AI-support) using the envelope-method. All radiographic images used in our study were anonymized and originated from patients who had given informed general consent for further use of their patient data within study frameworks.

After the allocation, each participant was confronted with 20 realistic, but virtual wrist trauma cases. All cases are based on real examples. The physicians received general information on each case, such as the patient’s age, gender, and a short description of how the injury occurred. In addition, they were given clinical findings and two projections of the corresponding wrist radiograph. The clinical information consisted of findings regarding swelling, pain, skin integrity, visible dislocation, sensory and motor function.

Our patient sample had an even gender distribution (10 females and 10 males) and an average age of 53 years (SD 19.88). Of the 20 cases, 10 displayed distal radius fractures whereas 10 exhibited non-fracture wrist trauma cases such as contusions or distorsions. For all cases, no other fractures than distal radius fractures were present. Within the fracture-cases, there was a variety in complexity ranging from obviously displaced fractures to rather subtle undisplaced fractures. A corresponding computer tomography (CT) scan was available for each case. The correctness of the diagnosis was checked threefold, once by a radiologist and twice by different hand surgery specialists. The AI-based model identified all fractures correctly, which means there were neither any false positives nor false negatives.

For our experiment, it was important that the physicians already encountered wrist-trauma-cases and were therefore familiar with the usual diagnostic procedure. They were asked to think of a situation in which they are alone in the emergency unit during a nightshift with no senior physician currently available.

**Fig. 2:**
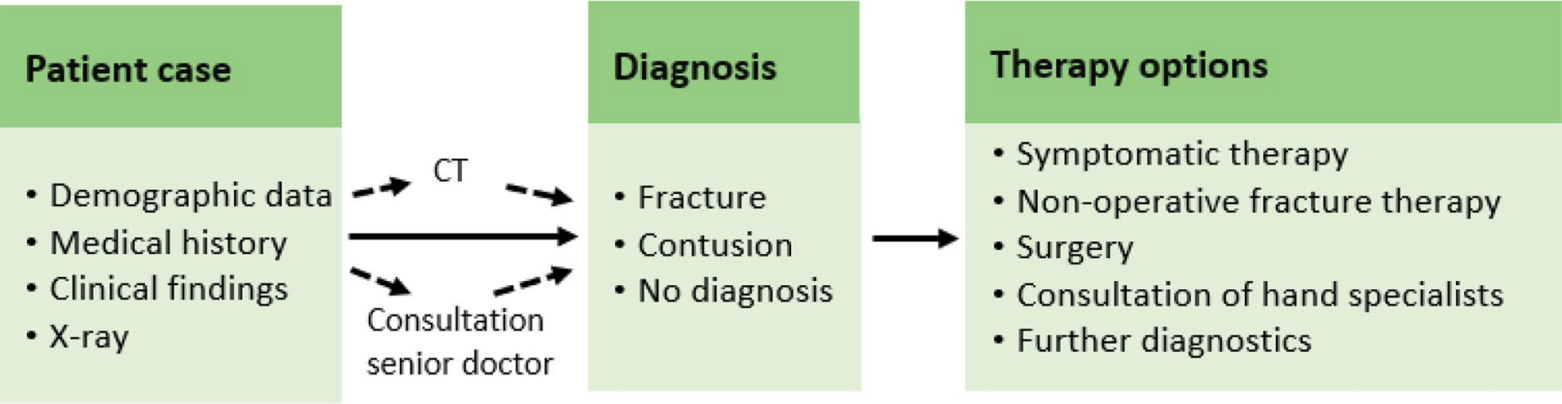
Questionnaire design

All 22 participants were given one trial case equally constructed as the following 20 cases to clear any questions and get them familiarized with the experiment setup. Each physician was asked to find the correct diagnosis and provide a treatment recommendation based on the available information. (Fig. 2).

For each case, four similar questions had to be answered in both groups. First, the participants needed to assess whether they had sufficient information available to provide a diagnosis and treatment or whether they wished to conduct a CT scan. Additionally, the physicians could decide to consult a senior physician, which meant that they were given the correct diagnosis by the experimenter to replicate the clinical setting. Afterwards, the participants made a diagnosis and decided on the therapy options. Lastly, they indicated how secure they felt about their diagnosis and treatment decisions. This sequence was repeated identically for all 20 cases.

### Outcome measurements

#### Primary outcome measurements

Firstly, we evaluated the physicians’ ability to make a diagnosis. We distinguished between instances where they did not require additional assistance and cases where they opted for support either through a CT scan—participants received an extra CT image of the scan—or consultation with a senior physician—participants were provided with the accurate diagnosis.

Secondly, we examined the diagnosis provided by the physicians. For each case, they were tasked with selecting one of three diagnosis options: “contusion”, “distal radius fracture” or “I don’t know”. The assessment of the diagnostic outcomes was contingent on the physicians' capability to discern between a fracture and a contusion.

The sensitivity and specificity statistics do not include the “I don’t know” responses. They are, however, included as a “false” answer in the mean diagnosis correctness statistics. Additionally, we conditioned the diagnosis outcome measure on the physicians’ ability to make a diagnosis. We did so because the physicians who received additional support from the CT or the senior physicians had a different information set than the ones who rendered the diagnosis by themselves.

#### Secondary outcome measurements

##### Therapy options

To create an authentic setting, we assessed the precision of physicians in selecting the appropriate therapy option. Five options were provided: symptomatic therapy (pain killers and cooling), non-operative fracture therapy (casting), surgery, consultation with hand specialists and further diagnostics. The choices “Consultation of a hand specialist” and “Further Diagnostics”, involving the performance of a CT scan, were consistently deemed incorrect answers, as they do not propose a suitable therapy option.

##### Confidence concerning diagnosis and treatment

After each case, the participants had to reflect on how confident they felt about their diagnosis and treatment. This was measured on an ordinal scale from 1 to 5, where 1 equaled very low confidence and 5 very high confidence.

##### Time

We analyzed the time it took the participants to solve the overall case-set. This was automatically recorded by the survey-tool.

##### Subjective and objective stress level

In addition to the questionnaire, we examined subjective and objective stress levels of our participants. To determine objective stress-levels we analyzed the heart rate and blood pressure of each participant, which can indicate stress as described by [18] and [19]. The *Polar H7 Heart Rate Sensor* and *Polar V800 Watch* were used to measure the heart rate (HR). To determine the “rest heart rate” we recorded the HR in the introductory phase of the test. The HR measured during the execution of the 20 cases was classified as “stress heart rate”. We used the software *KubiosHRV* to compute the average heart rates, see [20]. To measure blood pressure we used the blood pressure monitor *Omron M-300* which captured blood pressure every five minutes. To calculate the mean arterial blood pressure (MAP) we used the formula by DeMers and Daliah [21]:

$$\text{MAP} = \text{DP} + 1/3 (\text{SP} - \text{DP})$$

where DP and SP denote diastolic and systolic blood pressure, respectively. We differentiated between blood pressure before starting the questionnaire and during filling the questionnaire.

To measure subjective stress levels, we apply the State-Trait-Anxiety-Inventory (STAI), Form Y-1, devloped by Charles D. Spielberger, see [22]. All participants answered a questionnaire of 20 items in between the trial case and the first of the 20 cases, and after the last case.

### Statistical analysis

For each outcome measure, we calculated group means and unbiased standard deviations and used a t-test to examine whether the means of the two groups are statistically different. We defined a statistically significant difference by the t-test’s *p* value being smaller or equal to 0.05. A two-tailed distribution and homoscedastic variances of the two samples were chosen. We decided to do so by applying a rule of thumb that suggests choosing homoscedasticity in case the two sample variances being smaller than 4, which is the case for most of our outcome variables.

## Results

The results of the experiment are depicted in Table 2.

**Table 2:** Results

Outcomes	Group A (AI support)	Group B (no AI support)	*p* value
Primary outcomes			
Diagnosis w/o support	0.75 ± 0.13	0.52 ± 0.18	0.00
Additional CT-scan	0.14 ± 0.09	0.28 ± 0.15	0.02
Consultation senior doctor	0.11 ± 0.09	0.21 ± 0.26	0.25
Sensitivity	1.00 ± 0.00	0.96 ± 0.07	0.06
Specificity	0.99 ± 0.03	0.93 ± 0.14	0.17
No diagnosis	0.04 ± 0.07	0.07 ± 0.05	0.41
Mean diagnosis correctness (MDC)	0.95 ± 0.08	0.89 ± 0.10	0.08
Mean diagnosis correctness fractures	1.00 ± 0.00	0.96 ± 0.17	0.06
Mean diagnosis correctness contusions	0.91 ± 0.15	0.81 ± 0.17	0.16
Primary outcomes conditional measures			
Correct diagnosis|medical history	1.00 ± 0.00	0.92 ± 0.09	0.01
Specificity|medical history	1.00 ± 0.00	0.92 ± 0.13	0.04
Sensitivity|medical history	1.00 ± 0.00	0.96 ± 0.09	0.15
Correct DiagnosisExtra CT scan	0.72 ± 0.40	0.77 ± 0.20	0.76
Specificity|Extra CT scan	0.97 ± 0.08	0.88 ± 0.31	0.38
Sensitivity|Extra CT scan	1.00 ± 0.00	0.77 ± 0.11	0.40
Secondary outcomes			
Correct therapy	0.52 ± 0.12	0.50 ± 0.14	0.77
Confidence	3.86 ± 0.57	3.66 ± 0.65	0.45
Time (seconds)	1793 ± 512	1971 ± 515	0.43
STAI before test	29.42 ± 8.08	33.50 ± 10.73	0.32
STAI after test	28.83 ± 7.46	37.30 ± 11.58	0.05
HR before test (bpm)	76.00 ± 9.91	72.67 ± 12.78	0.51
HR after test (bpm)	74.50 ± 9.39	70.90 ± 12.34	0.45
MAP before test	101.9 ± 11.5	101.8 ± 11.4	0.99
MAP after test	98.05 ± 9.12	92.84 ± 6.98	0.15

Numbers are means; ± standard deviations

### Primary outcomes

We found that the physician’s ability to make a diagnosis without additional help by a CT or senior doctor is strongly affected by the use of the AI tool. The AI-supported group was able to make a diagnosis without support (no CT, no senior consultation) in 75% (SD = 0.13) of the cases, whereas the control group only made a diagnosis in 52% (SD = 0.18) of cases. This difference is statistically significant (*Diagnosis w/o support*, *P* = 0.003). The group without AI support chose an additional CT scan in 28% (SD = 0.15) of cases whereas the group with support only did so in 14% (SD = 0.09) of cases (*Additional CT-Scan*, *P* = 0.02). On average, the AI group also chose to consult a senior doctor less often, even though this difference is not significant (*Consultation senior doctor*; Group A: Mean = 0.11, SD = 0.09; Group B: Mean = 0.21, SD = 0.15; *P* = 0.25).

Regarding the ability to make a correct diagnosis, the use of the AI tool increased the sensitivity from 96 to 100% ($S{D}_{A} = 0,\, S{D}_{B} = 0.07, \, P = 0.06$) and the specificity from 93 to 99% ($S{D}_{A} = 0.03, \,S{D}_{B} = 0.14,\, P = 0.17$), however, neither of the statistics are significantly different. Also, if we included the “I don’t know” (*No Diagnosis*) responses as “false” answers, the ability to make a correct diagnosis increased on average: the mean correctness of the diagnosis increased from 89% (SD = 0.1) to 95% (SD = 0.08, *P* = 0.08) if all 20 cases were considered (*Mean Diagnosis Correctness*), from 96% (SD = 0.07) to 100 (SD = 0.0, *P* = 0.06) if only the fractures were considered (*Mean Diagnosis Correctness Fractures*), and from 81% (SD = 0.17) to 91% (SD = 0.15, *P* = 0.16) if only the contusions were considered (*Mean Diagnosis Correctness Contusions*). None of the increases in the means were statistically significant.

### Primary outcomes: conditional measures

Only considering the cases where the participants provided the diagnosis solely based on the medical history and x-ray (Correct Diagnosis|Medical history), we found that the AI group always made the right diagnosis, whereas the non-AI group only did so with a probability of 92% (SD = 0.09). The difference is significant (*p* = 0.01). Participants were more likely to identify a contusion correctly if they received support from the AI tool (Specificity|Medical history; Group A: Mean = 1, SD = 0; Group B: Mean = 0.92, SD = 0.13; *p* = 0.04). Regarding the correct identification of a fracture, we did not find a significant difference (Sensitivity|Medical history; Group A: Mean = 1, SD = 0; Group B: Mean = 0.96, SD = 0.09; *p* = 0.15). Lastly, only considering the cases where the participants chose to perform an additional CT scan, we did not find significant difference between the AI supported group and the control group (Correct Diagnosis|Extra CT Scan: *p* = 0.76; Specificity|Extra CT Scan = 0.38; Sensitivity|Extra CT Scan: *p* = 0.40).

### Secondary outcomes

*Subjective stress level*—We found the AI-supported group to be significantly less stressed compared to the control group (*STAI after Test*; Group A: Mean = 28.83, SD = 7.5; Group B: Mean = 37.3, SD = 11.6; *p* = 0.05).

The differences regarding the remaining secondary outcome measurements were not statistically significant.

## Discussion

Distal radius fractures are the most frequent fractures in human and account for 17.5% of all fractures in adults [23]. Radiographic images are the cornerstone of finding the correct diagnosis in wrist trauma patients. Usually, radiographs are interpreted by humans, mostly radiologists. However, several studies indicate a high number of diagnostic errors in radiograph interpretation with real-time error-rates of up to 3–5% in daily practice. [1–4]

In contrast, the literature indicates that AI-enabled fracture detection models can match expert accuracy in detecting fractures [9]. Nevertheless, widespread adoption in clinical practice remains limited. A potential barrier to embracing these technologies could be the absence of evidence in existing research connecting the use of AI tools to valuable benefits in terms of physician workload, patient clinical outcomes, or economic impacts.

We found that the AI tool increased the physicians’ diagnostic accuracy. The mean sensitivity rose from 96 to 100% and the mean specificity increased from 93 to 99% when employing the AI tool. However, the increase is not statistically significant. Notably, in situations where physicians made decisions independently, without additional support from a CT scan or a senior physician, the accuracy of the diagnosis significantly improved from 92 to 100%. Comparing our findings to the literature, [24] investigated the use of an AI tool by physicians in detecting of appendicular skeletal fractures and found that that AI assistance improved the sensitivity by 8.7% points, while the specificity increased by 4.1% points.

The increase in sensitivity and specificity we observed has potentially crucial implications for clinical routines. Firstly, the AI tool can facilitate more precise diagnosis in emergency units. Moreover, in settings with a lack of experts, such as family or general practitioners, the AI tool can serve as initial assistance for making further clinical decisions that might be challenging without the tool. Furthermore, in emergency units, junior physicians often conduct the initial interpretation of radiographs and subsequently consult senior physicians. The AI tool can enhance this process.

Our findings indicate that the presence of the AI tool led to a 50% reduction in the need for additional CT scans. This holds particular significance in clinical settings, as it substantially diminishes the requirement for additional CT scans. Consequently, this not only minimizes radiation exposure for patients but also results in cost savings for emergency units.

While not statistically significant, our study shows a 5% boost in diagnostic confidence among physicians using AI, being in line with Hoppe et al.'s [25] findings that AI support makes physicians feel more secure in evaluating X-rays. This increased confidence could decrease reliance on further diagnostics like CT scans or senior consultations and aid in training junior doctors through ongoing feedback.

Additionally, AI assistance led to a 9% reduction in time to complete tasks, offering potential benefits such as lighter physician workloads, shorter patient wait times, and less crowded emergency rooms. However, it is noteworthy that the observed difference is not statistically significant, as also found by Hoppe et al. [25]. This could be attributed to a potential lack of confidence in the AI tool among physicians, which might lessen with more familiarity, prolonged exposure or more regular use of the AI tool.

Physicians using AI also reported 23% lower stress levels, suggesting AI integration could ease the strain on doctors, particularly beneficial amid increasing demands on juniors.

Our findings position AI as a supportive tool rather than a replacement, aligning with previous studies that view AI as a "second reader" or “safety net”. [8] Our study has the potential to lay the foundation for further research on AI in clinical settings.

Our study has several limitations. Physicians had different levels of clinical and radiological experience. Notably, those using the AI tool generally had greater clinical experience. With random group assignment, controlling for experience disparities was not feasible. This likely influenced the results, as greater experience with radiological interpretation tends to improve diagnostic accuracy, an aspect not accounted for in our evaluation. The study involved a relatively small number of participating physicians, and the experiment's duration was limited. The external validity of the study could be enhanced with more participants.

Additionally, being under observation might have influenced physician behavior, potentially leading to more meticulous case analysis than usual—a phenomenon referred to as the “Hawthorne effect.”. [26]

Finally, accurately mirroring the complexity of real-life clinical settings poses significant challenges, which renders our findings more suggestive than definitive.

## Conclusion

We demonstrated that employing an AI tool for diagnosing distal radius fractures could lead to less unnecessary additional CT scans, reduced consultations of senior doctors, enhanced accuracy in the diagnosis when physicians make the diagnosis without additional help and potentially lower stress-levels.

## Data Availability

No datasets were generated or analysed during the current study.
